# Non-MHC immunity genes do not affect parasite load in European invasive populations of common raccoon

**DOI:** 10.1038/s41598-023-41721-1

**Published:** 2023-09-21

**Authors:** Aleksandra Biedrzycka, Maciej K. Konopiński, Marcin Popiołek, Marlena Zawiślak, Magdalena Bartoszewicz, Agnieszka Kloch

**Affiliations:** 1 Institute of Nature Conservation, Polish Academy of Sciences, Al. Mickiewicza 33, 31-120, Kraków, Poland; 2 Department of Parasitology, Faculty of Biological Sciences, University of Wrocław, Przybyszewskiego 63/67, 51-148 Wrocław, Poland; 3 Ekspertyzy Przyrodnicze, Szpitalna 2, 66-436 Słońsk, Poland; 4 Faculty of Biology, University of Warsaw, Miecznikowa 1, 02-089 Warszawa, Poland

**Keywords:** Environmental impact, Evolutionary genetics, Genetic variation, Invasive species

## Abstract

Understanding the evolutionary mechanisms behind invasion success enables predicting which alien species and populations are the most predisposed to become invasive. Parasites may mediate the success of biological invasions through their effect on host fitness. The evolution of increased competitive ability (EICA) hypothesis assumes that escape from parasites during the invasion process allows introduced species to decrease investment in immunity and allocate resources to dispersal and reproduction. Consequently, the selective pressure of parasites on host species in the invasive range should be relaxed. We used the case of the raccoon *Procyon lotor* invasion in Europe to investigate the effect of gastrointestinal pathogen pressure on non-MHC immune genetic diversity of newly established invasive populations. Despite distinct differences in parasite prevalence between analysed populations, we detected only marginal associations between two analysed SNPs and infection intensity. We argue that the differences in parasite prevalence are better explained by detected earlier associations with specific MHC-DRB alleles. While the escape from native parasites seems to allow decreased investment in overall immunity, which relaxes selective pressure imposed on immune genes, a wide range of MHC variants maintained in the invasive range may protect from newly encountered parasites.

## Introduction

Invasive species disrupt ecological communities, drive population declines and species extinctions. Parasites may affect the invasion success of their hosts through their effect on fitness and thus on host population growth and stability^1–4^. According to the enemy release hypothesis, newly established populations of non-native species harbour fewer enemies (pathogens and parasites) in the introduced range compared to the native range. As a result population regulation is reduced and spatial expansion accelerates^5^. According to the EICA hypothesis, i.e. evolution of increased competitive ability such an escape from parasites should favour introduced species that can decrease investment in immunity and allocate resources to dispersal and reproduction, thereby enhancing their invasive potential^6^. Along with leaving pathogens behind, the selective pressure on host species should be lower in invasive than in native ranges. However, non-native populations may come into contact with native species and accumulate novel pathogens, gaining high infection intensities over a relatively short time (e.g.^7^). If this is the case, ability to fight those infections is crucial to a successful invasion.

A recent meta-analysis indicated that parasite success is limited by the host's genetic diversity^8^. Since pathogens exert strong selection pressure on their hosts, immunity-related genes are presumed to be under selection due to host–pathogen coevolution^9, 10^. However, such relationships have been rarely studied in invasive populations. The importance of immune gene diversity is supported by significant associations between specific alleles and susceptibility to infections. This has been reported in many species; however, most of these examples are based on the components of the adaptive immunity with a wide range of studies focussing on MHC genes^11–15^. The role of other immunity genes in coping with parasite infection is often studied using a small number of Toll-like receptor (TLR) or cytokine genes^16–22^ although several hundred genes are involved in innate and acquired immune response. Genes evolving under balancing selection maintain high levels of diversity. MHC genes, which are usually characterised by dozens to hundreds of allelic variants segregating in natural populations^15^ or TLRs^23^, may play an important role in the immune defence of invasive species^24^. This is because the high level of the invasive populations' immune standing genetic variation, is crucial for their pathogen resistance (reviewed by^25^). The innate immune response is responsible for recognizing the pathogen and then initiating the acquired immune response^26, 27^. The PRRs (pattern recognition receptors) are the first line of pathogen detection^28^). Cytokines are signalling molecules capable of triggering and modulating the immune response, are the crucial link between innate and adaptive immunity. They mediate in removing the larval stages of gastrointestinal parasites and indirectly regulate the functioning of the mechanisms of the acquired immune response and enhancing the response from Th2 lymphocytes^27, 28^. Nevertheless, the type of selection shaping the diversity of innate immune genes usually leads to maintaining limited number of genetic variants. In case of positive or purifying selection that is found to shape diversity of many classes of innate immunity genes^29^ low frequency variants may be lost due to bottleneck occurring when the invasive population is established^16, 24^. Therefore, we may expect different roles of different groups of immune related genes in the response to the pathogen pressure in an invasive environment.

In the present study, we used the case of the raccoon *Procyon lotor* invasion in Europe to investigate the associations between a large set of immune genes and gastrointestinal parasites in newly-established invasive populations. The raccoon is a medium-sized carnivore whose native distribution in North America extends from southern Canada to Panama^30^. The first successful introduction in Europe occurred in Germany in the 1930s with a limited number of individuals^31^. Recently, approximately 1,000,000 raccoons were estimated to be living in Germany, and the range of the species in Europe has extended to the west, east and south of the invasion core^32, 33^. Native populations of raccoon are a reservoir of numerous viral (rabies virus, canine distemper virus), bacterial (*Leptospira* spp., *Francisella tularensis*) and parasitic (*Baylisascaris procyonis*, *Toxoplasma gondii*) pathogens^34–36^, but invasive populations are relatively mildly infected with parasites^37–39^ in comparison with native raccoon populations and other carnivore species in the invasive range^40–44^. Multiple genetic clusters detected throughout the European range of the raccoon provide the evidence for multiple independent introductions^45, 46^. Previously reported associations between specific MHC-DRB resistance and susceptibility alleles and digenean parasite *Isthmiophora melis* infection suggest an important role of this gene region in providing local adaptation to intestine parasites^37^. Specific infection-associated alleles were partly fixed in populations established from different introductions, causing extreme infection-level differences. This finding underlines the role of standing genetic variation, at least in MHC loci, in shaping host-parasite relationships in invasive populations and provides empirical support that functional genetic variation may be responsible for differences in invasion success^37^. In the present work, we analysed four previously studied invasive raccoon populations, varying considerably in the prevalence of intestinal parasites. The first population was located at the invasion core in central Germany and possessed relatively high levels of genetic variation, possibly due to mixing with divergent raccoon populations^46^. Two others, one from eastern Germany and another from western Poland, were located towards the invasion front and form the eastern edge of the raccoon invasion. The fourth population, located in northern Czech Republic, was established putatively from individuals which escaped from captivity was separated from the remaining ones and was characterised by a significantly lower level of genetic diversity^16, 47^.

The objective of this study was to test if a wide range of single nucleotide polymorphisms (SNPs) located in non-MHC immune related genes, in addition to MHC-DRB locus diversity, shape the levels of gastrointestinal infections in invasive raccoon populations. We aimed to reveal the main genetic determinants of infection in invasive raccoon populations. We expected that the associations between infection levels and genetic diversity may differ for the MHC gene, where high levels of genetic variation were retained in invasive populations and non-MHC immunity genes were characterised by relatively lower numbers of genetic variants. Our second objective was to discuss our findings in light of the EICA hypothesis. Invasive raccoon populations have lower parasitic infection levels in comparison to both the native raccoon populations and other carnivores that share a habitat with the raccoons in the invasive range. The putative release from enemies could allow invaders to outcompete native species and invest less into immune responses. The lowered parasite pressure might have resulted in relaxed selection pressure. Then visible as lack of associations between immune genetic variants and intestinal parasites is expected. The relaxation in selection pressure should be visible mainly in terms of costly innate immunity.

## Methods

### Ethics statement

Our study involved the raccoon as a study species but no animal was captured or killed specifically for the purpose of the project. No experiments involving live animals were performed for the purpose of this particular study. Most of the carcasses used in the study were obtained courtesy of hunters controlling this species in accordance to national hunting laws in all three countries (Poland: The Hunting Act of 13th October 1995 https://isap.sejm.gov.pl/isap.nsf/download.xsp/WDU19951470713/U/D19950713Lj.pdf, Germany: https://ljv-sachsen-anhalt.de/?wpdmpro=jagdzeiten-in-sachsen-anhalt, https://www.ljv-mecklenburg-vorpommern.de/Jagd-in-M-V/Jagd-und-Schonzeiten/ and the Czech Republic: The Hunting Act of 27th November 2001 https://www.zakonyprolidi.cz/cs/2001-449). Additional carcasses were obtained from road accidents. For these reasons, there were no experimental protocols to be approved by a named institutional and/or licensing committee. All methods were carried out in accordance with relevant guidelines and regulations and are reported in accordance with ARRIVE guidelines.

### Sample collection

**Figure 1:**
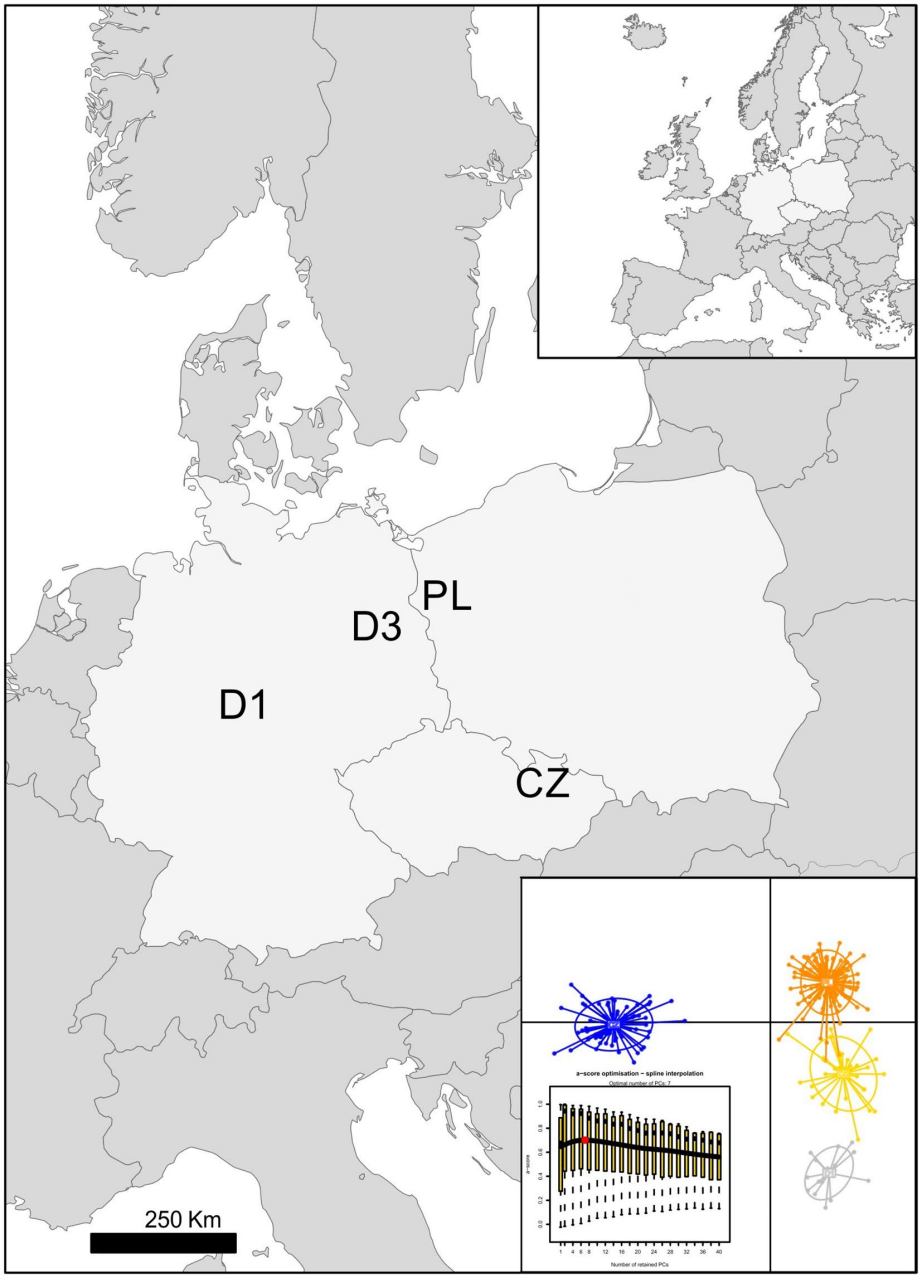
Geographical locations of invasive raccoon populations and the DAPC scatterplot of genetic differentiation across individuals resulting from DAPC analysis performed on 197 SNPs located in exon fragments of immune genes. The number of seven retained PCs chosen using a-score procedure. The map was generated using ArcGIS software by Esri. ArcGIS and ArcMap are the intellectual property of Esri and are used herein under license. Copyright Esri. All rights reserved. For more information about Esri software, please visit http://www.esri.com.

We collected 240 raccoon carcasses from the European part of the introduced range of the species: 116 from Poland, 18 from D1 population and 34 from D3 in Germany, and 72 from CZ population in Czech Republic. The sampling took place between 2012 and 2017. The localities of the sampling sites are shown in Fig. 1. The carcasses were obtained from hunters culling raccoons as part of game management activities to reduce the number of invasive species populations in all three countries and collected as road-killed individuals. No animal was killed or used in order to conduct this study. We collected only animals that were proven to be killed not longer than several hours earlier, with no signs of decomposition. The tissue samples for DNA analyses were collected as ear fragments or other tissues preserved in ethanol. The carcasses were kept frozen at − 20 °C prior to dissection.

### Parasite screening

The detailed procedure describing parasite screening is described in^37^. Briefly, each animal was sexed, weighed and measured for body length during dissection. Based on body length, weight and month of death, animals were classified as juvenile or adult. The whole alimentary tract was examined and macroscopically screened for the presence of helminths. All the isolated helminths were rinsed, counted and preserved in 70 or 90% ethyl alcohol.

### DNA extraction and immune diversity screening

DNA was extracted using the NucleoSpin Tissue Kit (Macherey and Nagel, Dueren, Germany). We selected a set of 246 non-MHC genes, proved to be associated with helminth infection, most of which are involved in innate immunity^48^. To perform targeted resequencing of the exon fragments for selected genes, we developed Molecular Inversion Probes (MIPs^49^). A detailed protocol describing MIP design and subsequent resequencing in studied individuals is described in^47^. The list of genes where analysed SNPs are located is given in Supplementary Table S1, online.

### Population structure

To visualise the population structure displayed at the SNP loci and identify discrete population clusters for inter-population description of parasite infections and defining the cofactor for models used in associations analysis, we used discriminant analysis of principal components (DAPC) implemented in the R package *adegenet*^50^. Multivariate analyses are the method of choice when the assumptions of Hardy–Weinberg and linkage equilibrium within populations are violated, which might be the case for genes under selection. First, using the function *find.clusters*, we identified the number of clusters that best reflects the genetic structuring in the data without a prior assignment of samples to given populations, using BIC scores (Bayesian Information Criterion^50^). A two-step procedure was used to establish an optimal number of principal components used in the DAPC. First, maximum number of principal components was established with a cross-validation function *Xval*.*Dapc*. The cross validation was performed as follows: 14,000 replicates were used to establish the optimal value from group mean, the maximum number of tested PCs was 80 and the training set comprised of 80% of observations, The resulting number of PCs was used to perform the DAPC. The number of PCs was further refined using α-score (*a-score* function). The resulting number of the optimal PCs was used in a second round of the DAPC. The results of the DAPC were plotted using the *scatter* function, with a population as a grouping factor.

### Association analysis

Of the initial file containing 254 individuals genotyped in 1772 SNPs, we removed individuals with over 20% of ungenotyped loci. Next, we removed variants with minor allele frequency MAF < 0.05, variants not in Hardy–Weinberg equilibrium, and those in lineage disequilibrium (threshold value of r^2^ > 0.65). Selection can affect HWE, but there can also be also other reasons for disequilibrium. We decided to remove loci not in HWE, following recommendations for association analysis (PLINK manual for example). This step is advised as such variants may result from genotyping errors. Furthermore, using R we checked for correlations between SNPs situated in different chromosomes, as it was not possible to test for associations between them using LD. From each group of loci correlated with r > 0.7 we randomly selected one locus for further analyses. These rather conservative filtering criteria resulted in 197 SNPs located in exon fragments of 115 immune related genes (Supplementary Table S1, online) that were used in the association analysis in R.

Due to the relatively low number of infected individuals, we could not test for the associations between parasite load and genetic variants for all parasite species detected in the analysed samples. We focused on the presence/absence of parasites rather than the intensity of the infection, and we considered higher parasite taxa (class/phylum) rather than species. The exception was the most frequent fluke *I. melis,* which infected 38% of racoons, and we tested for associations between genotypes and infection intensity only for this parasite.

The effects of SNPs on the presence/absence of parasites (*I. melis*, *Digenea, Cestoda* and *Nematoda*) were tested in R package SNPassoc^51^ under five genetic models (codominant, dominant, recessive, overdominant and log-additive) with genetic cluster membership, identified by the DAPC, included as a cofactor. Effect sizes were estimated using odds ratio implemented in the package SNPassoc.

The SNPs' effects on infection intensity with *I. melis* were tested in R using zero inflated models implemented in R package glmmTMB^52^ with population (genetic cluster) as random effect. We used negative binomial rather than Poisson distribution to control for the overdispersion. Effect size was estimated using the coefficient of determination R2 calculated separately for fixed effects and the whole model using r.squared GLMM function from the MuMIn package^53^. In several models we encountered a problem of complete separation what resulted in glmmTMB function failing to estimate model parameters. The separation occurred when the rarest genotype (present in fewer than 5 individuals) was observed in just one of four studied populations. Thus, were removed such genotypes before fitting the models what solved the separation problem. In the case of six SNPs we could not apply this procedure, as these SNPs had only two genotypes so after removing the one causing separation, only one genotype would have remained. In 10 models, the glmmTMB function returned convergence problem due to non-positive-definite Hessian matrix. Following the package tutorial we removed the zero-inflated part resulting in flat mixed-effect models with negative binomial distributions. In all models, we applied the most conservative correction for multiple comparisons which is the Bonferroni correction.

The prevalence differed between populations (Table 1). Parasite prevalence in PL and D3 populations was considerably higher than in the two remaining ones, so we fitted models two types of models: (1) using all data and (2) using only data from the two clusters with the highest prevalence (PL and D3).

**Table 1:** Gastrointestinal parasite prevalence by population. All species except *I. melis* had a prevalence < 10% and were summarised as higher taxa.

	CZ	D1	D3	PL
n	72	18	34	116
*I. melis*	6.06	0	42.86	60.33
Digenea	7.58	11.11	42.86	63.64
Cestoda	6.06	0	65.71	9.09
Nematoda	6.06	55.56	11.43	3.31

*n* number of individuals sampled per population, prevalence given in %

A common problem in association analysis is the difficulty to detect variants of low effects (low odds ratio) or in low frequencies in a population^54^. Such variants usually require large sample sizes which is hard to achieve in a study of free-living species. Thus, to check a detectability threshold of our study, we performed power analysis using epi.sscc function from the package epiR^55^ following the example 8.18 from Woodward^56^.

## Results

### Population structure

The DAPC analysis performed on 197 SNPs with seven PCs retained (Fig. 1) revealed clear differentiation between raccoons from four locations. Individuals from the introduction core in Central Germany and Czech Republic formed two separate clusters. Two other more closely related clusters were created by individuals from Eastern Germany and Western Poland, the relatively smaller distance between them is in accordance with the raccoon expansion from Germany towards the east (Fig. 1).

### Parasite prevalence and intensity

A total of 240 raccoons (132 males and 108 females) were screened for parasite infection. The analyses revealed a total of 15 parasite species taxa representing four main groups: *Digenea*, *Cestoda*, *Nematoda* and *Acanthocephala* (Table 1). The most prevalent parasite was trematode I*. melis, which* infected nearly 40% of the hosts. Two species of tapeworms were present in approximately 8% of the hosts. Interestingly, the racoon-specific *Baylisascaris procyonis* was found in only 9 hosts. Other nematode species, similar to Acantocephala, were found in less than 1% of hosts (Supplementary Table S2a, online). We detected pronounced differences in the basic parasitological indices between analysed populations. The prevalence of infection was very different for raccoons from populations CZ and D1 than from PL and D3 both for *Digenea* (7.58% in CZ; 11.11% in D1; 42.86% in D3; 63.64% in PL, Table 1) and *Cestoda* infection (6.06% in CZ; 0% in D1; 65.71% in D3; 9.09% in PL, Table 1).

### SNPs–parasite associations

When we considered all four populations, we found a significant effect of SNP 26438_387 on infection intensity with the fluke I. melis (chi^2^ = 26.295, padj = 0.004, (Fig. S1 online). This SNP is located in GMNN gene that codes for geminin, DNA replication inhibitor. Individuals with genotype T/T had significantly higher parasite burden (301.55 flukes) compared with heterozygotes and homozygotes C/C (49.77 and 23.11 respectively). Among heterozygotes T/T we found two individuals of the highest parasite load (858 and 1042). Notably, those two individuals were clearly outliers, as the parasite load in other infected individuals did not exceed 480, and the mean number of flukes per infected host was 109. When the model was fitted without the outliers, the effect of SNP was no longer significant (chi^2^ = 9.3404, df = 2, p = 0.009; below the adjusted p = value threshold of 0.00025). We also found a significant effect of the SNP 194809_624, located in IL6ST; interleukin 6 signal transduction gene (chi^2^ = 22.874, df = 2, padj = 0.0021, Fig. S1 online) but again, the strong effect of the genotype GG can be explained by the fact that it was present in a single individual with second-highest parasite load (890 flukes). When this individual was removed, the association of SNP 194809_624 with *I. melis* infection intensity was no longer significant (chi^2^ = 2.8609, df = 2, p = 0.091). These same effect was observed for both SNPs when two populations with the lowest parasite load were removed from the models.

We found no significant associations between risk of infection (presence/absence of a given parasite) and genotype for any parasite group tested (*I. melis*, *Digenea*, *Cestoda*, *Nematoda*) under five analysed genetic models. The exact p-values for each SNP and each model are given in (Supplementary Table S2a online). The tables presenting odds-ratio tests for each SNP and each parasite are given in Supplementary Information 1–11 online.

When we rejected two populations with the lowest parasite load (D1 and CZ), we detected one significant association after correcting for multiple analyses. The SNP located in NRCAM gene was associated with the prevalence of Nematoda infection under recessive model: in the infected group, individuals with the genotype TT constituted 57% compared to the other genotypes combined (CC and CT). In the non-infected group, they represented just 11% of individuals. Still, taking into account that the number of infected individuals was only 4 and 3 in D3 and PL, respectively, we consider this result as an artefact. The exact p-values are given in Supplementary Table S2b online. The tables presenting odds-ratio tests for each SNP and each parasite are given in Supplementary Information 1–11 online.

The power analysis (Fig. S2 online) showed generally for a given odds-ratio, higher power was achieved when infection rate was low. However, the generally satisfactory power of 80% for infection levels > 70% was achieved only when the odds-ratio was higher than 2.25. This suggest that in our sample size and infection levels, we were only able to detect relatively strong effects, and weaker effects might have been unnoticed.

## Discussion

In this study, we used a targeted sequencing approach to investigate how the associations between the non-MHC immunity gene diversity and gastrointestinal parasite infections are shaped in invasive populations of the raccoon. We studied four populations of invasive raccoons with different levels of parasite infection. In our previous study, we identified MHC-DRB alleles altering the probability of Digenean (*I. melis*). The presence/absence of MHC-DRB alleles in different populations reflected demographic process related to population establishment during invasion but, in turn, allowed for creating strong differences in population susceptibility to infection. Here we studied the importance of a wide range of non-MHC immunity genes in controlling gastrointestinal infections in invasive raccoon populations.

Studied populations varied considerably in the prevalence of *Digenea* and *Cestoda*, with D1 and CZ populations having an order of magnitude lower prevalence than D3 and PL populations. Nevertheless, using association analyses, we did not identify SNPs that would explain such sharp differences in pathogen prevalence. Notably, the only significant association with the infection intensity could be contributed to the presence of three individuals with extra high parasite load. Although statistically valid, this result is hard to interpret biologically. After removing the outliers, the effect of SNPs was no longer visible. Thus, it is difficult to conclude whether those SNPs really affect susceptibility or rather the individuals with enormous parasite load had other traits making them prone to such an abundant infection.

The most common intestinal parasite group was Digeneans (with *I. melis* being the most frequent parasite). Its prevalence in D3 and PL was 0.412 and 0.69, respectively (Table 1) and was much lower in CZ and D1 populations (0.042 and 0.059, respectively; Table 1). According to the available data *I. melis* is widespread in Europe and uses a wide range of definitive vertebrate hosts typical for environment inhabited by raccoon genetic clusters studied here^57^. The trematodes have a complex life cycle, and the raccoon gets infected by ingesting infected snails, fish or amphibians^58^. Since the intermediate hosts may disperse on their own, it is not likely that the observed differences in infection levels between clusters are due to the differential occurrence of *I. melis* in the environment.

The second most frequent parasite group infecting racoons was Cestodes. *Atriotaenia incisa* is a parasite of badgers, and Mesocestoides are common in medium and large carnivores; both have also been described in invasive raccoon populations^59^. Similarly to trematodes, an infection occurs after consuming infected intermediate land-dwelling hosts, such as rodents. To the contrary, nematodes detected in the current study have a simple lifecycle, where infection occurs directly via contact with faeces shredded by an infected carnivore. Thus, the infection risk with trematodes and cestodes is associated with feeding habits, while infection with nematodes occurs during direct or indirect contact with other carnivores or other vertebrate species. Interactions of raccoons with other species in the invasive range may differ from the native range, resulting in a lower infection prevalence. Moreover, generally low parasite load in invasive raccoons was previously described^60^, which supports the enemy release hypothesis.

Several factors may contribute to the differences in parasite load between populations. Each of them presents a different demographic history and forms well separated geographical units. Although potentially connected, German populations (D1 and D3) located in the continuous range of invasive raccoon populations are characterised by different frequencies of mitochondrial haplotypes and were probably established by separate introductions^46^. The Polish population was formed by the expansion of raccoons towards the east and is genetically and geographically close to German population D3. A case such as this, we can expect that, along with genetic variants transferred during population spread, the transmission of *I. melis* also occurred, although there is a prerequisite of the presence of intermediate hosts in the expansion range. The CZ population was established from separate introduction, putatively from individuals from a different source population where different host-parasite genetic associations might have evolved. It is possible considering the intraspecific variability of this trematode species^57^. Therefore individuals that established CZ population might have evolved specific immune protection to the *I. melis* present in the native range, while such associations did not arise in native populations that acted as a source for German and Polish populations. There are also habitat differences between the studied genetic clusters. Both D3 and PL populations inhabit marshes, wetlands and lake regions with a high abundance of intermediate hosts^58^, while D1 occupies dry woodland habitat. These differences may contribute to the differential prevalence of *Digenea* between D1 vs. D3 and PL populations but not between CZ vs. D3 and PL as the Czech population also inhabits the lake region and wetlands (Table 1).

Although it is hard to define ecological factors explaining why two genetic clusters (D1 and CZ) present very low infection levels, genetic factors may play a major role in shaping these differences. As we previously detected, the MHC-DRB allele found in CZ cluster, but not in others, was associated with resistance to *I. melis*. It could suggest that adaptive immune response, linked mainly to MHC loci variation, could play a primary role in fighting *I. melis* infection. MHC genes that exhibit extremely high polymorphism maintained by balancing selection may be more important in coping with infections in recently established invasive populations than other immune genes. A wide range of genetic variants maintained by balancing selection creates relatively high standing genetic variation that enables invasive populations to respond quickly to novel environmental conditions^37, 61^. The release from enemies believed to occur during invasion helps invaders outcompete native species. According to the increased competitive ability (EICA) hypothesis, invaders are predicted to invest less in immune responses^5^, but this lowered response is expected mainly for costly innate immunity. Innate defences are mobilised quickly and are effective against novel pathogens but impose higher metabolic and inflammatory costs^62^ and may be downregulated in highly active and dispersing organisms^63^. On the other hand, the adaptive immune response is specific to various pathogens but acts effectively only after primary contact with the pathogen. In the case of invading species that can get rid of pathogens during the invasion and do not encounter many novel enemies in the new range, adaptive immune response, that is associated with lower cost, may act more effectively. This process was observed in invasive edge populations of cane toads^64^ or in expansive populations of neotropical thrushes that exhibited lower infection levels and lowered inflammation compared to native populations^65^. The level of gastrointestinal infections in invasive raccoon populations is relatively low compared to native species. It suggests the "escape" from higher pathogenic load and potential decrease in the innate immune response. The studied 197 SNPs are located in non-MHC, mostly innate immunity genes. In the case of the suppressed innate immune response, the associations between studied SNPs and parasite load would rather not be established, especially in relatively recently formed populations. The lack of associations suggests that analysed SNPs of non-MHC genes do not show signs of pathogen-induced selection. At least they are not under a divergent selection in invasive raccoon populations, as we did not find associations explaining between-populations differentiation in infection level. Although it is established that specific groups of innate immunity genes follow divergent evolution pattern^66, 67^, a short time since the establishment of the invasive population may be not enough to observe any clear selection pattern. On the contrary, a wide range of specific functional MHC variants transferred to the new range^16^ may constitute sufficient basis of genetic variation on which pathogen mediated selection could act.

## Conclusions

Our study shows that non-MHC innate immunity gene diversity does not play a crucial role in fighting gastrointestinal infections in invasive raccoon populations. This observation is in line with the increased competitive ability hypothesis that relates the downregulation of innate immunity with higher dispersal and survival in novel environmental conditions. At the same time, we suggest that invasive populations may acquire immune response via adaptive immunity as specific MHC-DRB variants regulate gastrointestinal infection levels in studied invasive populations.

### Supplementary Information

Supplementary Figure S1.

Supplementary Figure S2.

Supplementary Table S1.

Supplementary Table S2a.

Supplementary Table S2b.

Supplementary Legends.

Supplementary Information 1.

Supplementary Information 2.

Supplementary Information 3.

Supplementary Information 4.

Supplementary Information 5.

Supplementary Information 6.

Supplementary Information 7.

Supplementary Information 8.

Supplementary Information 9.

Supplementary Information 10.

Supplementary Information 11.

## Data Availability

The VCF file containing SNP genotypes used in this work have been deposited on GitHub (https://github.com/konopinski/raccoon). All the data necessary to reproduce the analysis presented in the manuscript are provided as supplementary material.
