# Supplementary figures and images for: Non-MHC immunity genes do not affect parasite load in European invasive populations of common raccoon
Source: Sci Rep. 2023 Sep 21;13:15696. doi: 10.1038/s41598-023-41721-1 (PMC10514260; doi:10.1038/s41598-023-41721-1)

4 populations

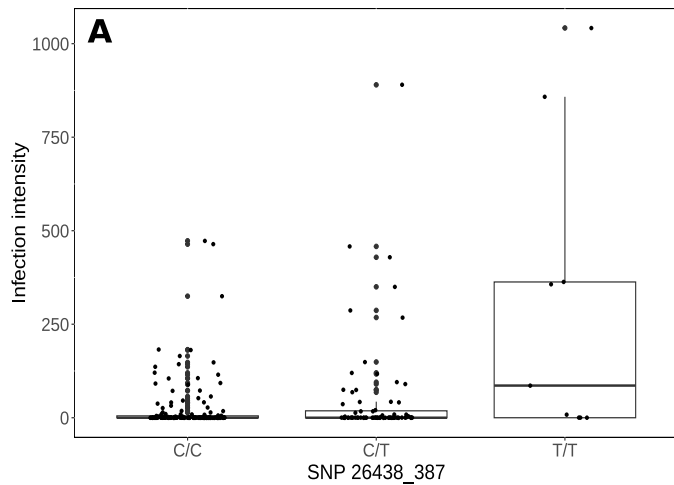

2 populations

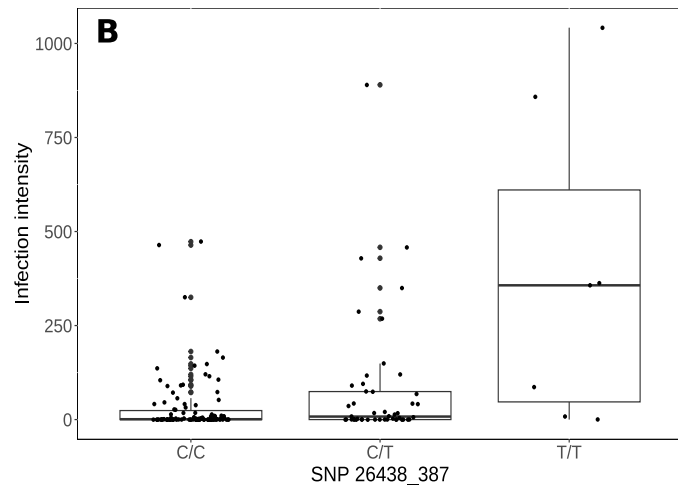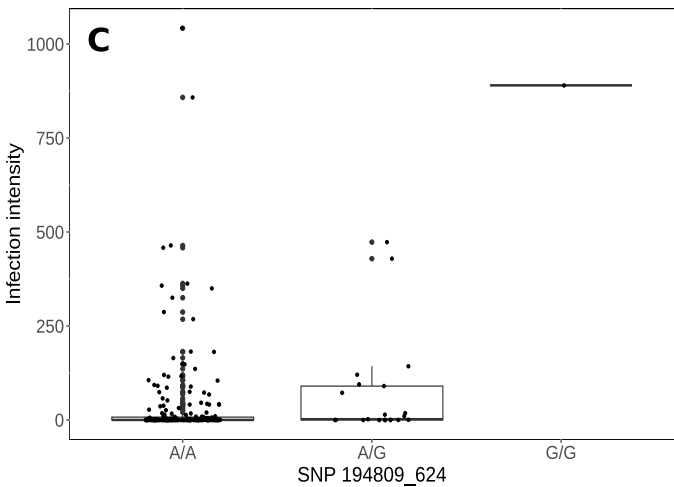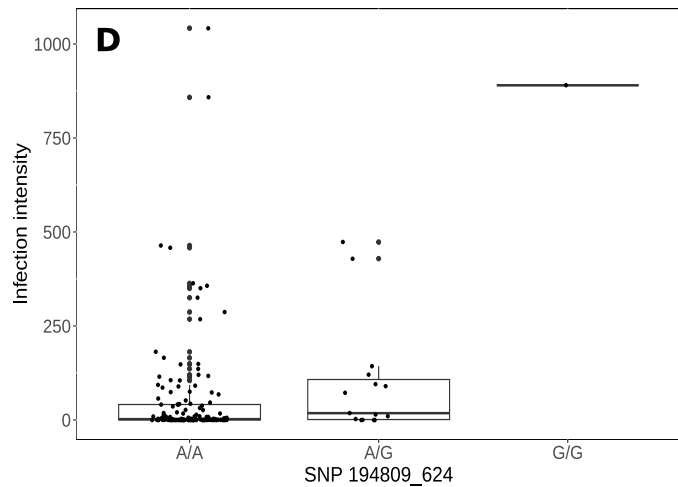

Supplement: Supplementary file 1 — Supplementary Figure S1. [file 41598_2023_41721_MOESM1_ESM.pdf]

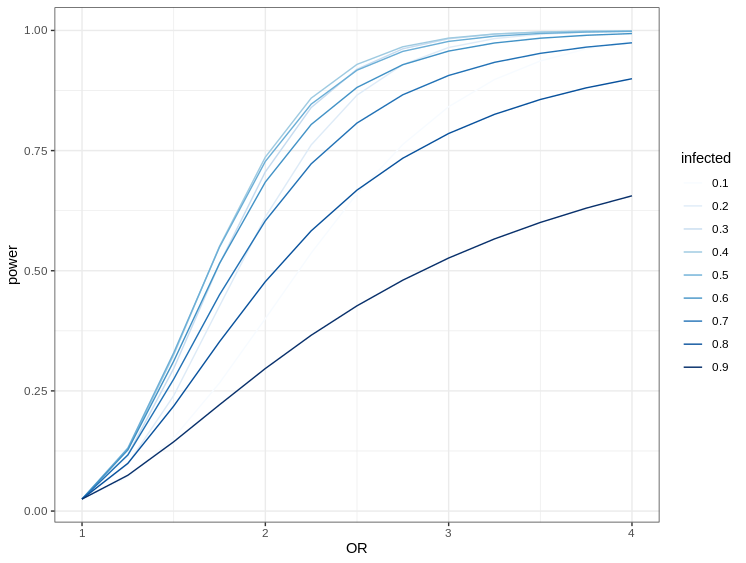

Supplement: Supplementary file 2 — Supplementary Figure S2. [file 41598_2023_41721_MOESM2_ESM.png]
