# Supplementary material for: Non-MHC immunity genes do not affect parasite load in European invasive populations of common raccoon
Source: Sci Rep. 2023 Sep 21;13:15696. doi: 10.1038/s41598-023-41721-1 (PMC10514260; doi:10.1038/s41598-023-41721-1)
Supplement: Supplementary file 6 — Supplementary Legends. [file 41598_2023_41721_MOESM6_ESM.docx]

Fig S1. Effect of SNPs affecting parasite load as indicated by zero-inflated mixed effect models. Box represent mean and 25 ane 75% percentile. The dots represent observations.

Fig S2. Power of the association tests (parasite presence/absence vs genotype) depending on the odds ratio and fraction on infected individuals within one of the compared genotypes.

Supplementary Table S1. The list of analysed immune-related genes.

Supplementary Table S2a. SNPs – parasite associations. Models calculated for four populations.

Supplementary Table S2b. SNPs – parasite associations. Models calculated for four populations.
